# Effective interventions for gaming disorder: A systematic review of randomized control trials

**DOI:** 10.3389/fpsyt.2023.1098922

**Published:** 2023-02-06

**Authors:** Yuzhou Chen, Jiangmiao Lu, Ling Wang, Xuemei Gao

**Affiliations:** ^1^Faculty of Psychology, Southwest University, Chongqing, China; ^2^Key Laboratory of Cognition and Personality, Ministry of Education, Southwest University, Chongqing, China

**Keywords:** gaming disorder, intervention, randomized controlled trials, group counseling, systematic review

## Abstract

**Objective:**

To identify effective intervention methods for gaming disorder (GD) through a rigorous assessment of existing literature.

**Methods:**

We conducted a search of six databases (PubMed, Embase, PsycINFO, CNKI, WanFang, and VIP) to identify randomized controlled trials (RCTs) that tested GD interventions, published from database inception to December 31, 2021. Standardized mean differences with 95% confidence intervals were calculated using a random effects model. Risk of bias was assessed with the Risk of Bias 2 (RoB 2) tool.

**Results:**

Seven studies met the inclusion criteria. Five interventions were tested in these studies: group counseling, craving behavioral intervention (CBI), transcranial direct current stimulation (tDCS), the acceptance and cognitive restructuring intervention program (ACRIP), and short-term cognitive behavior therapy (CBT). Four of the five interventions (the tDCS was excluded) were found to have a significant effect on GD. The results of the quality assessment showed that the included studies had a medium to high risk in the randomization process and a medium to high risk of overall bias.

**Conclusion:**

Rigorous screening identified that four interventions are effective for GD: group counseling, CBI, ACRIP, and short-term CBT. Additionally, a comprehensive review of the literature revealed that improvements could be made in the conceptualization of GD, experimental design, sample representativeness, and reporting quality. It is recommended that future studies have more rigorous research designs and be based on established standards to provide more credible evidence to inform the development of GD interventions.

## 1. Introduction

Electronic games have gradually become one of the most popular forms of entertainment. Excessive playing of such games, however, can harm physical and mental health and lead to gaming disorder (GD). A recent meta-analysis (1) estimated the global prevalence of GD at 8.5% for males and 3.5% for females. Of all the global regions, Asia showed the highest prevalence (6.3%), followed by North America (3.6%), Oceania (3.0%), and Europe (2.7%). The children and adolescent age group had the highest prevalence (6.6%).

The term “gaming disorder” refers to uncontrollable or persistent online or offline gaming behavior. Other terms that describe this kind of addictive behavior include gaming addiction (2), pathological gaming (3), and problematic gaming (4). This review uses the term gaming disorder (GD) throughout. As a behavioral addiction, GD does not require the intake of any substances, yet it shares psychological characteristics, such as heightened arousal, craving, and tolerance, with substance addiction and other behavioral addictions (5). Psychological problems associated with GD include increased aggression (6), depression (7), anxiety (8), loneliness (9), and impulsivity (10). Many young people become immersed in gaming for substantial periods of time, often neglecting their studies and living isolated lives. The American Psychiatric Association (APA) has proposed that internet gaming disorder (IGD) is potentially an addictive disorder (11), while the new International Classification of Diseases-11 (ICD-11) includes GD in its chapter on behavioral addiction (12).

The causes of GD could be explained from different perspectives. Behaviorally, it is speculated that GD is caused by an imbalanced sensitivity of the behavioral activation system (BAS) and behavioral inhibition system (BIS) (13, 14), such that persistent gaming behaviors are cued or triggered easily but they are not effectively inhibited. Neurologically, GD appears to be associated with the abnormal functioning of the striatum, an important part of the brain’s reward processing system (15). Dynamic switching between ventral and dorsal striatal connections is a fundamental neural characteristic of GD (16). Physiologically, GD is possibly associated with abnormal dopamine release and reception (15). The dopamine release for GD is similar in magnitude to that for drug abuse (15) and a low level of D2 receptors in the striatum is correlated with years of gaming (17). Psychologically, maladaptive cognitions are thought to be the key cause of GD (18); these could lead to more positive evaluations of the virtual world and over-reliance on the game, and ultimately, to gaming disorder.

According to the above studies on the causes of GD and other relevant literature, existing interventions for GD can be broadly divided into four categories: behavioral therapy (19–22), electrotherapy (23, 24), pharmacotherapy (25–27), and psychotherapy (28–36), with some programs using a combination of these interventions (37–44). Specifically, behavioral therapy exerts its effect on the behavioral mechanism of GD; for example, by reducing the association between game-related stimuli and the game-players responses to them. This therapy focuses on the behavior itself, aiming to feel, identify with, substitute, or change the gaming behavior. Unlike behavioral therapy, psychotherapy focuses on regulating cognition and mental state. It helps individuals to correct their maladaptive cognition and regain psychological support, which ultimately induces changes in behavior. Some psychotherapies, such as cognitive behavioral therapy (CBT), are combined with behavioral intervention. Electrotherapy focuses on the fundamental mechanism of behavior and cognition. Electrical stimulation is claimed to change the neurophysiological features of the brain and body, which manifests as psychological and behavioral changes. Regarding pharmacotherapy, because there is no medicine used to treat GD directly, pharmacological drugs like atomoxetine, bupropion, and methylphenidate are used to treat the comorbidity of GD, such as attention deficit and hyperactivity disorder (ADHD) and depression, to relieve the severity of GD symptoms. Comparing the effects of the different interventions, one research study (45) found that combining pharmacotherapy with CBT or multi-level counseling (MLC) may be the most effective treatment for teenagers with internet addiction (IA) or IGD, while another study (46) also indicated that combined interventions were more effective for IA. Table 1 presents the main types of GD interventions along with their theoretical principles and operative points. A list of relevant studies with their full titles is shown in Supplementary Table 1.

**TABLE 1:** Characteristics of gaming disorder (GD) interventions.

Categories	Interventions	Principles	Operation points	References
Behavioral therapy	Arts therapy	To arouse free association through art (sand play, music, dance, etc.) to regulate emotions.	No details provided.	Ryu (22)
	CET	Diminish conditioned associations by systematically pairing them in a treatment setting.	No details provided.	Zhang et al. (21)
	PE	Reduce stereotypical behaviors through feeling motor stimulations.	Six 90-min sessions that included stretching, warm-up, aerobic exercises, and feedback.	Hong et al. (19)
	SRC	Reduce reward sensitivity using stimulus-response compatibility training.	Participants pressed buttons in either compatible or incompatible conditions. The intervention lasted for 15 days.	Zheng et al. (20)
Electrotherapy	tDCS	Stabilize changes in gamma and beta EEG activity.	Electrodes were placed over the left and right DLPFC. Each session contained two 20-min stimulations (2.0 mA) separated by a 20-min interval.	Lee et al. (24)
	Electro-acupuncture	Mobilize the body’s disease-fighting factors and bioelectricity by stimulating acupuncture points.	The acupuncture points received electrical stimulation, 15 min every other day for a total of 4 weeks.	Tang et al. (23)
Pharmcotherapy	ATM	Selectively inhibit norepinephrine reuptake.	ATM was started at 10 mg/day and increased to 60 mg/day during the first 2 weeks of treatment according to individual symptoms.	Park et al. (27)
	Bupropion SR	Block the reuptake of norepinephrine and dopamine.	Bupropion SR was started at 150 mg/day during the first week and then increased to 300 mg/day over the following 5 weeks.	Han et al. (26)
	MPH	Decrease dopamine D2 receptor availability in the striatum.	The initial dosage was 18 mg/day. This was adjusted during the first 4 weeks based on changes in clinical symptoms and weight.	Han et al. (25)
Psychotherapy	CBI	Enhance cognitive control and reduce the salience of gaming-related cues.	CBI was conducted weekly in group sessions on a specific topic; 6 sessions in total.	Zhang et al. (30)
	CBT	Monitor thoughts, feelings, and behaviors associated with games.	The intervention was a 12-session course with two sessions per week. Each two sessions had a specific theme, Each session lasted 45 min.	Li et al. (29)
	Family therapy	Treat psychological problems through family interaction.	Each family created exercises designed to improve family cohesion and worked on their new interactions for more than 1 h/day and 4 days/week.	Han et al. (31)
	Group counseling	Improve mental health conditions by interacting with group members.	Group counseling consisted of 6 sessions with 6 themes, lasting for 3 weeks at 2 h per session.	Huang et al. (33)
	MET	Intrinsic motivation is the real power for change.	Therapy was based on four stages of motivation: contemplation, preparation, action, and consciousness.	Pontes and Griffiths (69)
	Mindfulness	Self-regulate attention and adopt a particular orientation toward one’s experiences.	No details provided.	Sharma et al. (32)
	Narrative therapy	Guide participants to rebuild positive stories, thereby awakening their inner power for change.	The procedure consisted of listening to participants’ stories, identifying unique outcomes, and externalizing the problems.	Graham (35)
	Reality therapy	Solve problems in realistic and reasonable ways; emphasize the present and the future.	The therapy had 6 × 2-h sessions with six topics specific to the characteristics of IGD.	Yao et al. (34)
	VRT	Regulate neurobiological imbalance in the limbic system.	Each session included relaxation, simulation of a high-risk situation, and sound-assisted cognitive reconstruction; 8 sessions in total.	Park et al. (28)

CET, cue exposure therapy; PE, physical exercise; SRC, stimulus-response compatibility; tDCS, transcranial direct current stimulation; ATM, atomoxetine; SR, sustained release; MPH, methylphenidate; CBI, craving behavioral intervention; CBT, cognitive behavioral therapy; MET, motivational enhancement therapy; VRT, virtual reality therapy; EEG, electroencephalogram; DLPFC, dorsolateral prefrontal cortex; IGD, internet gaming disorder.

Despite the large number of intervention approaches developed over the past decade, as yet there are no authoritative guidelines for what makes an effective GD intervention. Besides the complexity of GD itself, we suggest that there are two main reasons for this. First, some studies have confused the concepts of IA and GD, employing the idea that IA interventions should also apply to GD. An updated meta-analysis from 2022 (45) explored the efficacy of treatments for children with IA/GD, while an integrative review (47) assessed the effectiveness of psychological interventions for IA and/or GD. Additionally, a later meta-analysis (46) indicated the most effective intervention for IA was combined method but did not distinguish between the specific types of IA. IA is an umbrella term for various types of internet-based behavioral addiction (48), including social media addiction, short video addiction, cyber-sexual addiction, gambling addiction, and gaming addiction. The Internet is a medium that is only used to engage in these addictive behaviors. IA is therefore not equal to GD and interventions to treat IA may not apply to GD. As such, it is not appropriate to put the two concepts together or to simply focus on the IA intervention itself without distinguishing its subcategories.

Second, there might be fewer studies that provide compelling evidence for GD intervention. According to the Oxford Center for Evidence-Based Medicine (OCEBM), randomized controlled trials (RCTs) provide the highest level of evidence (49) and numerous systematic reviews have specified that to provide the most credible evidence, only RCT studies should be used. However, only a small proportion of GD intervention studies have used an RCT design. This could be because RCT procedures are difficult to implement with GD groups or because GD interventions are not yet sufficiently developed. Furthermore, research approaches like interviews (50), case studies (51, 52), one-group pretest-posttest designs (38, 53, 54), and pretest-posttest control group designs (31, 34, 39, 55–57) are essential for feasibility analysis and useful for designing full-scale RCTs, but they may not be adequate for explaining the intrinsic effects of interventions. Some RCT studies have used a clinical or supportive control group, but this can only show a comparative effect, not the effect of the intervention itself (29, 58). Based on the above concerns, this study aimed to identify the most effective interventions for GD through a rigorous screening process.

## 2. Methods

### 2.1. Eligibility criteria

The criteria followed the PICOS principle (participants, interventions, comparators, outcomes, and study design) set out in the PRISMA guidelines (59). PICOS is a formatted retrieval method based on evidence-based medicine (EBM) that helps researchers to gather clinical evidence.

The inclusion criteria were: (1) Participants: diagnosed with GD or game-related addiction through scales or clinical criteria; (2) Interventions: the experimental group received a complete and systematic intervention (e.g., based on information about the objective, form, frequency, and duration of the intervention); (3) Comparators: the control group was in a non-active condition (e.g., received no-intervention or placebo); (4) Outcomes: the measurement of the severity of game-related addiction was included; (5) Study design: RCTs.

The exclusion criteria were: (1) Participants: IA but not game-related addiction, or studies that did not specify the type of IA; (2) Interventions: the experimental group did not receive a complete and systematic intervention (e.g., paradigm-based studies, or those based on natural recovery or one-shot methods); (3) Outcomes: studies did not provide quantitative indicators of intervention effects; (4) Other: non-experimental or incomplete studies.

### 2.2. Search strategy

We searched for studies in the PubMed, Embase, PsycINFO, CNKI, WanFang, and VIP databases published from inception to December 31, 2021. The search strategy specified a combination of search terms that had GD and intervention in the title or abstract: (Game OR Gaming) AND (Addicti* OR Compulsive OR Dependence OR Problematic OR Excessive OR Pathological OR Disorder OR Repeated OR Overuse* OR Maladaptive) AND (Intervention OR Treat OR Therapy OR Training OR Workshop OR Psychotherapy OR Pharmacological OR Program OR Curriculum). The search was limited to publications written in English and Chinese. Reference lists of eligible studies were manually searched to identify additional relevant studies. The full search strings for each database can be found in Supplementary Table 2.

### 2.3. Study selection

All citations were retrieved and screened according to PRISMA criteria (60). First, duplicate studies were removed. Second, two reviewers (YC and JL) screened the titles and abstracts, retaining those for which full-text screening was required. Two reviewers (YC and JL) screened the studies according to the eligibility criteria and extracted the data from the studies that were judged to be eligible. A third reviewer (LW) resolved any disagreements between the assessments of the two independent reviewers.

### 2.4. Data extraction

The data from the included studies were extracted into a descriptive summary table. The following information was extracted: author/year, country, sample, mean age, intervention type, duration of intervention, frequency of intervention, diagnostic method, outcome variables, and results.

### 2.5. Quality assessment

Two reviewers (YC and JL) assessed the quality of the included studies using the Risk of Bias 2 (RoB 2) tool (61). The tool assesses five domains of bias (randomization process, deviation from intended interventions, missing outcome data, measurement of the outcome, and selection of the reported results). Each domain has several signaling questions with five alternative answers: yes (Y), probably yes (PY), probably no (PN), no (N), and no information (NI). Based on the reviewer assessments, the risk of bias for each domain was divided into one of three grades: low risk of bias, some concerns, or high risk of bias. Any discrepancies were resolved by the third reviewer (LW).

## 3. Results

### 3.1. Study selection

**FIGURE 1:**
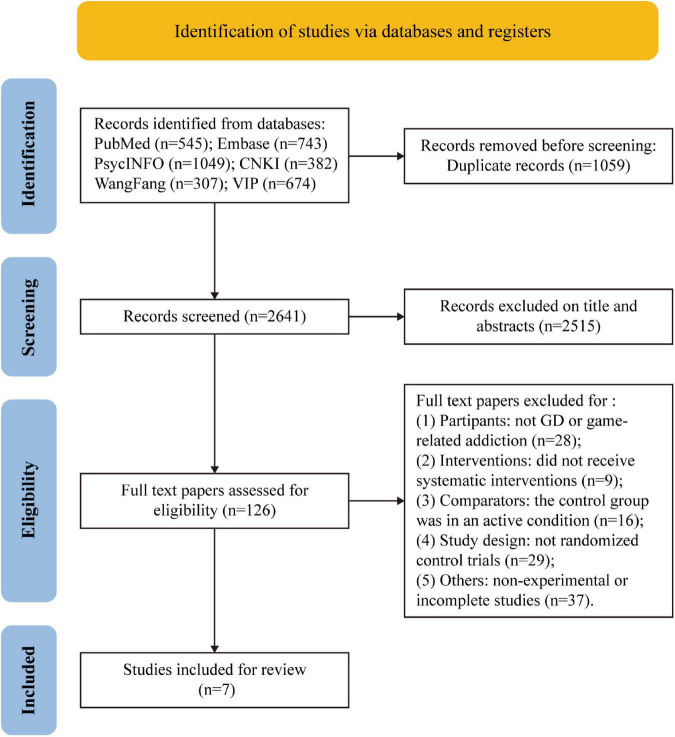
PRISMA flow diagram of the study selection procedure.

In total, 3,700 studies were identified from the databases (Pubmed = 545, Embase = 743, PsycINFO = 1,049, CNKI = 382, WanFang = 307, and VIP = 674). After removing 1,059 duplicates, 2,641 studies remained. Of these, 2,515 were excluded after the title and abstract screening, leaving 126 studies for full-text screening. Of these, 119 studies were excluded for the following reasons: (1) Participants: not GD or game-related addiction (*n* = 28); (2) Interventions: were not complete and systematic (*n* = 9); (3) Comparators: the control group was an active condition (*n* = 16); (4) Study design: not RCTs (*n* = 29); (5) Other: non-experimental or incomplete studies (*n* = 37). Finally, seven studies were retained for further analysis (Figure 1).

### 3.2. Study characteristics

#### 3.2.1. Participants

The characteristics of the included studies are shown in Table 2. Four of the studies were conducted in China (30, 33, 62, 63), while the other three were conducted in Korea, Spain, and Germany, respectively (24, 43, 64). The seven included studies tested a total of 332 participants, 174 in the experimental groups and 158 in the control groups. The number of participants included in the studies was 27, 32, 24, 40, 26, 40, and 143, respectively. Seven studies comprised both adolescent and adult samples (24, 30, 33, 43, 62–64), while four studies comprised only adult samples (24, 30, 33, 64). Three studies did not report the age of the participants (43, 62, 63), although two studies recruited the participants from middle school (62, 63), and the other comprised 16–19 years old teenagers (43).

**TABLE 2:** Characteristics of the included studies.

References	Country	Sample	Mean age (SD)	Type of intervention	Duration	Frequency	Diagnostic method	Outcome measures	Results
Huang et al. (33)	China	EG = 17 CG = 10	EG = 21.00 (1.00) CG = 21.00 (1.00)	EG: GC (IGC) CG: non-active	3 weeks	6	CGAI	CGAI, SAS, BDI, SES, CQ, game time	EG had lower post-test scores for the CGAI, SAS, BDI interventions, and the negative self dimension of the CQ compared to the CG.
Qiao (63)	China	EG = 16 CG = 16	NR (middle school students)	EG: GC (ACT) CG: non-active	4 weeks	8	OGAQ	OGAQ, AAQ-II, CFQ, SCS	EG had decreased OGAQ, AAQ-II, CFQ scores, and increased SCS scores after intervention; while the CG only showed an increase in the AAQ-II.
Du (62)	China	EG = 12 CG = 12	NR (junior high students)	EG: GC (CBT) CG: non-active	9 weeks	18	IGAS	IGAS, SS	EG had lower post-test scores for the IGAS compared to the CG. Also a decrease in the IGAS and SS scores within the EG.
Zhang et al. (30)	China	EG = 23 CG = 17	EG = 21.91 (1.83) CG = 22.00 (1.90)	EG: CBI CG: non-active	6 weeks	6	CIAS	CIAS, weekly gaming, behavioral and fMRI data for craving	EG had decreased IGD severity, weekly gaming hours, and cue-induced craving, enhanced activation in the anterior insula and decreased insular connectivity in the lingual gyrus and precuneus compared to the CG.
Lee et al. (24)	Korea	EG = 14 CG = 12	EG = 23.07 (5.78) CG = 25.33 (8.94)	EG: tDCS CG: non-active	5 days	10	DSM-5	EEG, IAT, VAS, BIS-11, BDI, BAI, SST	EG had no significant change in behavioral and psychological results. Gamma power in the left parietal region was decreased in the EG compared with the CG (the post-test was 1 month after the intervention).
Kochuchakkalackal Kuriala and Reyes (43)	Spain	EG = 20 CG = 20	NR (16–19 years old teenagers)	EG: ACRIP CG: non-active	5 weeks	10	IGDS-SF9, DSM-5	IGDS-SF9, PBW	For the EG, the post-test scores of the outcome variables showed a significant difference compared with their pre-test scores, in contrast to the GP.
Wölfling et al. (64)	Germany	EG = 72 CG = 71	EG = 26.20 (8.66) CG = 26.20 (6.94)	EG: ST-CBT CG: non-active	15 weeks	15	AICA-S AICA-C	AICA-S, AICA-C, GAF, SCID-I and -II, BDI-II	Both groups decreased in AICA, time spent, and BDI. EG exerted a larger effect.

EG, experimental group; CG, control group; SD, standard deviation; NR, not reported; GC, group counseling; IGC, interpersonal group counseling; ACT, acceptance and commitment; CBT, cognitive behavior therapy; CBI, craving behavioral intervention; tDCS, transcranial direct current stimulation; ACRIP, acceptance and cognitive restructuring intervention program; ST-CBT, short-term cognitive and behavior therapy; RCT, randomized control trial; CGAI, computer gaming addiction invention; OGAQ, Online Game Addiction Questionnaire; IGAS, Internet Game Addiction Scale; CIAS, Chen Internet Addiction Scale; DSM-5, Diagnostic and Statistical Manual of Mental Disorders (5th ed.); IGDS-SF9, Internet Gaming Disorder Scale—nine-item short-form; AICA, Assessment of Internet and Computer Game Addiction; SAS, Self-Rating Anxiety Scale; BDI, Beck Depression Inventory; SES, Rosenberg Self-Esteem Scale; CQ, Cognition Questionnaire; AAQ-II, Acceptance Action Questionnaire-II; CFQ, Cognitive Fusion Questionnaire; SCS, Self-Compassion Scale; SS, Shyness Scale; IAT, Young’s Internet Addiction Test; VAS, Visual Analog Scale; BIS-11, Barratt Impulsiveness Scale-11; BAI, Beck Anxiety Inventory; SST, Stop-Signal Task; PBW, Ryff’s Psychological Well-Being Scale; GAF, Global Assessment of Functioning; SCID, Structured Clinical Interview for DSM-IV.

#### 3.2.2. Interventions

All the included studies were RCTs with a non-active control group. Five different intervention methods were assessed. Three studies assessed group counseling (33, 62, 63) with three different themes (interpersonal interaction, acceptance and commitment, cognition and behavior), while the other four assessed a craving behavioral intervention (CBI) (30), transcranial direct current stimulation (tDCS) (24), an acceptance and cognitive restructuring intervention program (ACRIP) (43), and a short-term cognitive behavior therapy (CBT) (64). The mean duration of the seven interventions was 6.7 weeks and the duration of the individual interventions varied from 3 to 15 weeks; the mean frequency of training was 10.4 sessions and the individual interventions varied from 6 to 18 sessions.

#### 3.2.3. Diagnostic methods

The studies differed in their diagnostic methods, with each using a different method to screen the GD participants: Computer Gaming Addiction Invention (CGAI) (65), Online Game Addiction Questionnaire (OGAQ) (66), Internet Game Addiction Scale (IGAS) (67), Chen Internet Addiction Scale (CIAS) (68), Internet Gaming Disorder Scale—nine-item short-form (IGDS-SF9) (69), and Assessment of Internet and Computer Game Addiction (AICA) (70). One study was screened by a clinically experienced psychiatrist based on the DSM-5 (11). Although we use the term GD to refer to game-related addictive behaviors, different definitions of GD were used in the included studies because of the different measures used, namely “computer gaming addiction” (CGA) (33), “online game addiction” (OGA) (63), “internet game addiction” (IGA) (62), “internet gaming disorder” (IGD) (24, 30, 43), and “internet and computer game addiction” (ICGA) (64).

#### 3.2.4. Outcome measures

The primary outcome was the severity of GD measured by the diagnostic method of each study. Secondary outcomes were also measured. The most frequently found secondary outcomes were depression (24, 33, 64), anxiety (24, 33), cognition (33, 63), and game time (30, 33). Others included self-esteem (33), self-compassion (63), shyness (62), impulsivity (24), and psychological well-being (43). Other than the scales listed above, one study (30) used behavioral and fMRI methods to detect the level of craving for GD individuals, and another (24) adopted the Stop-Signal Task to find the change in executive function for the GD group after a series of interventions.

#### 3.2.5. Main findings

Table 2 shows the main results for each study. The effect of each intervention was assessed by the diagnostic method of each study using a pretest-posttest design. Of these, the group counseling, CBI, ACRIP, and short-term CBT interventions had a significant effect on decreasing the severity of GD (30, 33, 43, 62–64), while the tDCS intervention had no significant effect on behavioral and psychological indicators of GD (24). Based on the secondary outcomes, IGC (interpersonal group counseling) and short-term CBT significantly reduced depression in GD individuals (33, 64), IGC and ACT (acceptance and commitment) significantly reduced the maladaptive cognition of GD individuals (33, 63), and IGC and CBI significantly reduced the number of hours spent gaming for the GD group (30, 33). Furthermore, in GD individuals, IGC significantly reduced anxiety (33), ACT significantly increased self-compassion (63), CBT significantly reduced shyness (62), and ACRIP significantly increased psychological well-being (43). The CBI study also adopted fMRI and found significant changes in brain activation (30).

### 3.3. Risk of bias assessment

**FIGURE 2:**
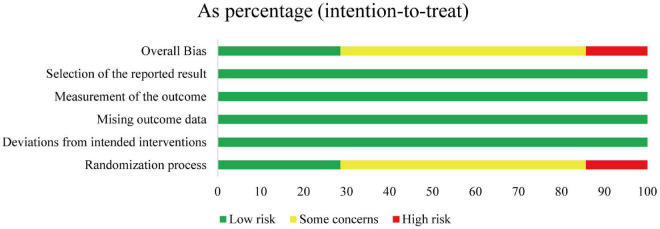
Risk of bias summary.

**FIGURE 3:**
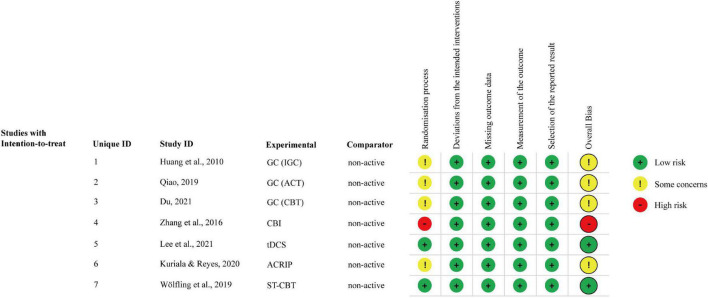
Risk of bias for each included study.

Overall, regarding the randomization domain, the seven included studies were all rated as medium to high risk (Figure 2). Specifically, one study showed a high risk of bias based on the willingness of participants to be assigned into groups (30). Four studies failed to report the randomization details (33, 43, 62, 63). The included studies showed a low risk of bias for the other four domains of bias: deviations from the intended process, missing outcome data, measurement of the outcome, and selection of the reported results Figure 3.

## 4. Discussion

The purpose of this study was to identify effective interventions for gaming disorder (GD) and issues relating to the current GD interventions. A systematic search of the available literature found seven studies that met the inclusion criteria. Five types of intervention were assessed in these studies: group counseling, CBI, tDCS, ACRIP, and short-term CBT. Of these, group counseling, CBI, ACRIP, and short-term CBT were found to significantly reduce the severity of GD, while tDCS was found to have no significant effect on GD (24).

### 4.1. Group counseling

Three of the included studies assessed group counseling (33, 62, 63), although each one differed in that they had a specific theme (interpersonal interaction, acceptance and commitment, cognition and behavior). The sample ages ranged from middle school students to young adults. Group counseling is a form of counseling that emphasizes the interpersonal communication of conscious thoughts, feelings, and behaviors using a here-and-now timeframe (71). A group of people of a similar age (from children to the elderly) meet to solve their problems. The most crucial point of this method is that group work creates an atmosphere where people can share their thoughts and communicate with each other, and explore their concerns while building empathy through interpersonal support from others. For individuals with GD, one identified motivation for persistent gaming is the need to escape from real-life problems (72). Those with GD are frequently in need of support but they rarely seek it out of a wish to avoid painful issues surrounding self-awareness and its associated negative effects (73). Group counseling can be effective because it provides a place for an individual to face reality with the support of others, who can help the individual to discover the inner strength they need to handle their problems (71). It should also be noted that group counseling typically revolves around a theme. The specific content of this theme and how this meets the psychological needs of GD individuals is an important consideration when designing a treatment plan.

### 4.2. Craving behavioral intervention (CBI)

Craving is one of the most characteristic features of GD. In the DSM-5, the first GD criterion is a preoccupation with internet games (11). In the ICD-11, additional clinical features of GD include often experiencing urges or cravings to engage in gaming during other activities (12). Craving works as a trigger for the onset of gaming behaviors; in turn, gaming behaviors enhance craving responses in GD individuals (74). Since craving may significantly affect the formation and maintenance of GD (75), interventions that help individuals deal with craving may improve therapeutic outcomes and prevent relapse. In the study by Zhang et al. (30), CBI was conducted weekly in groups of eight to nine young adults. Similar to group counseling, each session had a specific theme: (1) perceiving subjective craving; (2) recognizing irrational beliefs regarding craving; (3) relieving craving-related negative emotions; (4) training in coping with cravings; (5) learning skills for coping with craving; (6) reviewing, practicing, and implementing skills. Mindfulness training was included in each session. It was found that CBI may exert its effects by enhancing executive control over gaming behaviors and reducing the salience of gaming-related cues, which play a vital role in recovery from GD.

### 4.3. Acceptance and cognitive restructuring intervention program (ACRIP)

Based on two fundamental theories, the cognitive-behavioral model and mindfulness theory (76), the main objectives of ACRIP are to reduce the symptoms of GD and improve the psychological well-being of participants (43). The cognitive-behavioral model of pathologic internet use (PIU) emphasizes the important role of cognition (18). Problems in the real world give individuals a distorted cognition of the virtual world. For example, they may think, “People treat me badly offline; the Internet is my only friend.” This type of maladaptive cognition worsens GD symptoms; thus, it is crucial to restructure the cognitions of those that suffer from GD. Next, the theory of mindfulness is about individuals engaging in a state of mind where they can think and behave mindfully (77); mindfulness teaches individuals how to observe their thoughts and accept themselves in the moment. By accepting and restructuring their cognition, individuals can then change their behaviors and align them to their cognitions. The ACRIP has eight modules, each with a specific topic: (1) introductory session: accustomizing; (2) freeing oneself from dysfunctional thoughts; (3) forging oneself to create positive vibes; (4) igniting and rebuilding friendships and relationship; (5) rekindling self-love, self-respect, and approval; (6) magnifying self-worth and independence; (7) enabling one’s control over the external world; (8) developing a friendly atmosphere where creativity is enhanced. The ACRIP was found to be effective for reducing GD symptoms and increasing psychological well-being in adolescents, regardless of the cultural differences among them.

### 4.4. Short-term cognitive behavior therapy (CBT)

The most central factor element of the cognitive-behavioral model of PIU is the presence of maladaptive cognitions (18). People with PIU present fundamental cognitive dysfunction in the form of specific maladaptive cognitions, with thinking such as, “I am worthless offline, but online I am someone.” These maladaptive cognitions are proximal contributory causes of behavioral disorders and are sufficient to cause other associated symptoms. The central tenet of CBT is that all behaviors are a result of how people view objects and are based on their thoughts and beliefs (18). CBT helps individuals to practice identifying maladaptive behavioral cognitions, substitute maladaptive cognitions for adaptive ones, and change their core beliefs. When a person changes their beliefs toward an object, their behavior will change accordingly. It has been suggested that CBT is an effective intervention for GD (46, 47, 78), and can help individuals to address maladaptive cognitions and social and behavioral deficits, and motivate them to change and reestablish alternative behaviors (79). One study in this review adopted a short-term CBT intervention for young adults with a 15-week structure (43), which had three phases and 15 sessions: (1) early phase (sessions 1–3); (2) behavior modification (sessions 4–12); (3) and stabilization and relapse prevention (sessions 13–15). The intervention was to be found effective in adolescents and young to middle-aged adults for decreasing the severity of GD and depression and reducing the time spent on games (80).

### 4.5. Issues and suggestions

Although this study was able to identify potentially effective GD intervention methods, our analysis revealed several points worth considering in future GD intervention studies. We summarize these below under four categories and provide corresponding suggestions:

#### 4.5.1. Conceptualization of GD

There are various types of internet-mediated behavioral addictions, such as gaming, sex, short videos, and gambling. The Internet is merely the medium for engaging with these myriad activities, and what matters more for designing effective interventions is the specific behavior being addressed. Individuals addicted to online gaming are not internet addicts; they are gaming addicts that use the medium of the Internet to engage in their addictive behavior (81). One study that assessed the degree of overlap between IGD and PIU found that only 6.67% of participants with PIU were also classified as having IGD, and less than half of IGD participants also met the criteria for PIU (82). Therefore, IA and GD appear to be related but distinct concepts (83, 84). However, many studies still confuse these concepts (45, 47, 85, 86), resulting in mistargeted interventions that may be ineffective. Although many of those with GD do game online, GD is not equivalent to IA. Moreover, GD has been categorized as a psychological and behavioral disorder by the World Health Organization (WHO) and the APA (11, 12). As such, specific directed interventions should be used to treat it. Therefore, we suggest that future studies could focus specifically on GD or IGD when developing intervention methods.

#### 4.5.2. Study design and reporting

We noted in the introduction that most GD intervention studies have not used an RCT design, probably because of the complex process. Although interview or case studies are essential for feasibility analysis and useful for designing a full-scale RCT, they may not be adequate for explaining the intrinsic effects of interventions; this is why this exploration of effective GD interventions only included RCTs. Even so, the risk of bias still exists in the RCTs reviewed here, especially regarding the randomization process. Of the seven included studies, four did not describe the method of randomization (33, 43, 62, 63), while one assigned participants based on their willingness (30). Such methodological decisions can introduce confounding factors that may influence the results of the intervention. We suggest that future GD intervention studies carefully design and perform experiments based on the quality assessment. Authors must also improve the quality of reporting and follow international standards, such as the Consolidated Standards of Reporting Trials (CONSORT) (87) and the Journal Article Reporting Standards (JARS) (88).

#### 4.5.3. Measures and definitions

We specified the severity of GD as the outcome indicator for this review. However, the outcome measures differed across all studies, with assessments using different measurement tools: the CGAI (65), OGAQ (66), IGAS (67), CIAS (68), DSM-5 (11), IGDS-SF9 (69), and AICA (70). This discrepancy may have caused heterogeneity among the studies, such that findings may be difficult to generalize. In addition, the GD participants were typically not recruited through clinical diagnosis. The authority and representativeness of outcome measures should thus be carefully considered. Because of this difference in measures, five different definitions of GD were used in the included studies, namely the CGA, OGA, IGA, IGD, and ICGA. Although these all refer to game-related addictions, differences exist between their conceptualizations. For instance, the CGA and ICGA emphasize specific gaming devices while the remaining three treat game addiction as a whole. We therefore recommend that future studies adopt standardized conceptualizations, such as either “GD” from the ICD-11 or “IGD” from the DSM-5.

#### 4.5.4. Other points

Two other points should also be considered. First, the total sample size of the included studies was relatively small (*n* = 332), and three studies had less than 30 participants (24, 33, 62). In future studies, researchers could aim to increase the sample sizes to gain adequate statistical power for detecting potential effects. Second, in some studies, the samples were unrepresentative. Two studies recruited only male participants (24, 64), one added a shyness level threshold to the GD group (62), and another recruited only participants with high motivation for the GD intervention (33). We suggest that future studies standardize recruitment without adding additional requirements, to reduce or eliminate experimenter effects (89).

Despite the strengths of this systematic review, this interpretation of our findings should acknowledge some limitations. First, due to the rigorous screening procedure, a limited number of studies (seven) were included in this review. However, this does not mean that the interventions included here are the only effective ones; many other methods are widely used in GD interventions (see Table 1) but were excluded from this review because there was not enough compelling evidence to include them. The results of this review should be considered as reference material for researchers looking to find the most appropriate type of intervention. Second, this study did not specify the exact tool to be used for the outcome measurement, which may have resulted in heterogeneity between studies. Future studies might consider adding assessment type as an additional screening criterion. Third, we did not perform a quantitative analysis because of heterogeneity related to the interventions, diagnostic measures, and diverse populations. We suggest that future studies might identify the most effective GD intervention using a network meta-analysis. Finally, we included studies only written in English and Chinese, and did not consider studies from other cultural backgrounds. A more comprehensive review could be performed to better understand the nature of GD interventions across different countries and cultures.

In conclusion, a rigorous search and screening procedure found compelling evidence that group counseling, CBI, ACRIP, and short-term CBT are effective interventions for GD. We also examined the current status of GD interventions and found that improvements could be made in conceptualizing GD, experimental designs, sample representativeness, and reporting quality. We recommend that researchers in this field aim to carry out more rigorous studies of GD interventions based on accepted standards to provide more credible evidence about their use.

## Author contributions

YC: conceptualization, methodology, software, investigation, formal analysis, writing – original draft and review and editing, and visualization. JL: software, validation, investigation, data curation, and writing – review and editing. LW: methodology, validation, and writing – review and editing. XG: writing – review and editing, supervision, project administration, and funding acquisition. All authors contributed to the article and approved the submitted version.
