# Supplementary material for: Effective interventions for gaming disorder: A systematic review of randomized control trials
Source: Front Psychiatry. 2023 Feb 6;14:1098922. doi: 10.3389/fpsyt.2023.1098922 (PMC9940764; doi:10.3389/fpsyt.2023.1098922)
Supplement: Supplementary file 1 [file Table_1.DOCX]

Table S1. Characteristics of GD interventions

| **Catagories** | **Interventions** | **Author, Year** | **Study titles** |
| --- | --- | --- | --- |
| Behavioral therapy | Arts Therapy | Ryu, 2018 | Application of Expressive Arts Therapy for Game Addiction Children |
|  | CET | Zhang et al., 2016 | Cue-induced behavioral and neural changes among excessive Internet gamers and possible application of cue exposure therapy to Internet gaming disorder |
|  | PE | Hong et al., 2020 | Effect of physical exercise intervention on mood and frontal alpha asymmetry in internet gaming disorder |
|  | SRC | Zheng et al., 2022 | Reduction of symptom after a combined behavioral intervention for reward sensitivity and rash impulsiveness in internet gaming disorder: A comparative study |
| Electrotherapy | tDCS | Lee et al., 2021 | Neuromodulatory Effect of Transcranial Direct Current Stimulation on Resting-State EEG Activity in Internet Gaming Disorder: A Randomized, Double-Blind, Sham-Controlled Parallel Group Trial |
|  | Electro-acupuncture | Tang et al., 2017 | Clinical effect of cognitive behavior therapy combined with electro-acupuncture in the treatment of internet gaming disorder |
| Pharmcotheropy | ATM | Park et al., 2016 | Effectiveness of atomoxetine and methylphenidate for problematic online gaming in adolescents with attention deficit hyperactivity disorder |
|  | Bupropion SR | Han et al., 2010 | Bupropion sustained release treatment decreases craving for video games and cue-induced brain activity in patients with Internet video game addiction |
|  | MPH | Han et al., 2009 | The effect of methylphenidate on Internet video game play in children with attention-deficit/hyperactivity disorder |
| Psychotherapy | CBI | Zhang et al., 2016 | Effects of craving behavioral intervention on neural substrates of cue-induced craving in Internet gaming disorder |
|  | CBT | Li et al., 2013 | The role of cognitive distortion in online game addiction among Chinese adolescents |
|  | Family Therapy | Han et al., 2012 | The effect of family therapy on the changes in the severity of on-line game play and brain activity in adolescents with on-line game addiction |
|  | Group Counceling | Huang et al., 2010 | Effects of interpersonal group counseling on college students with computer gaming addiction |
|  | MET | Pontes & Griffiths, 2015 | Internet gaming disorder: Application of motivational enhancement therapy principles in treatment |
|  | Mindfulness | Sharma et al., 2021 | Mindfulness-Based Interventions: Potentials for Management of Internet Gaming Disorder |
|  | Narrative Therapy | Graham, 2014 | Narrative therapy for treating video game addiction |
|  | Reality Therapy | Yao et al., 2017 | Combined reality therapy and mindfulness meditation decrease intertemporal decisional impulsivity in young adults with Internet gaming disorder |
|  | VRT | Park et al., 2016 | The effects of a virtual reality treatment program for online gaming addiction |

*Note*: CET = cue exposure therapy, PE = physical exercise, SRC = stimulus-response compatibility, tDCS = transcranial Direct Current Stimulation, ATM = atomoxetine, SR = sustained release, MPH = methylphenidate, CBI = craving behavioral intervention, CBT = cognitive behavioral therapy, MET = motivational enhancement therapy, VRT = virtual reality therapy, EEG = electroencephalogram, DLPFC = dorsolateral prefrontal cortex.
