# Supplementary material for: Effective interventions for gaming disorder: A systematic review of randomized control trials
Source: Front Psychiatry. 2023 Feb 6;14:1098922. doi: 10.3389/fpsyt.2023.1098922 (PMC9940764; doi:10.3389/fpsyt.2023.1098922)
Supplement: Supplementary file 2 [file Table_2.DOCX]

Supplement 2. Full search strings of databases

PubMed:

(Game[Title/Abstract] OR Gaming[Title/Abstract]) AND (Addicti*[Title/Abstract] OR Compulsive[Title/Abstract] OR Dependence[Title/Abstract] OR Problematic[Title/Abstract] OR Excessive[Title/Abstract] OR Pathological[Title/Abstract] OR Disorder[Title/Abstract] OR Repeated[Title/Abstract] OR Overuse*[Title/Abstract] OR Maladaptive[Title/Abstract]) AND (Intervention[Title/Abstract] OR Treat[Title/Abstract] OR Therapy[Title/Abstract] OR Training[Title/Abstract] OR Workshop[Title/Abstract] OR Psychotherapy[Title/Abstract] OR Pharmacological[Title/Abstract] OR Program[Title/Abstract] OR Curriculum[Title/Abstract])

Embase:

(game:ab,ti OR gaming:ab,ti) AND (addicti*:ab,ti OR compulsive:ab,ti OR dependence:ab,ti OR problematic:ab,ti OR excessive:ab,ti OR pathological:ab,ti OR disorder:ab,ti OR repeated:ab,ti OR overuse*:ab,ti OR maladaptive:ab,ti) AND (intervention:ab,ti OR treat:ab,ti OR therapy:ab,ti OR training:ab,ti OR workshop:ab,ti OR psychotherapy:ab,ti OR pharmacological:ab,ti OR program:ab,ti OR curriculum:ab,ti) AND [<1966-2021]/py AND ('article'/it OR 'article in press'/it) AND 'human'/de

PsycINFO:

TI ( (Game OR Gaming) AND (Addicti* OR Compulsive OR Dependence OR Problematic OR Excessive OR Pathological OR Disorder OR Repeated OR Overuse* OR Maladaptive) AND (Intervention OR Treat OR Therapy OR Training OR Workshop OR Psychotherapy OR Pharmacological OR Program OR Curriculum) ) OR AB ( (Game OR Gaming) AND (Addicti* OR Compulsive OR Dependence OR Problematic OR Excessive OR Pathological OR Disorder OR Repeated OR Overuse* OR Maladaptive) AND (Intervention OR Treat OR Therapy OR Training OR Workshop OR Psychotherapy OR Pharmacological OR Program OR Curriculum) ) Linked Full Text; Peer Reviewed; Publication Year: 1986-2021; English; Document Type: Journal Article
